# ﻿A new species of *Metopiellus* (Coleoptera, Staphylinidae, Pselaphinae) from the northern Colombian Amazon

**DOI:** 10.3897/zookeys.1108.76077

**Published:** 2022-06-23

**Authors:** Gianpiero Fiorentino, Maria C. Tocora, Sebastian Ramirez

**Affiliations:** 1 Department of Biological Sciences, New Jersey Institute of Technology, Dr. Martin Luther King Jr Boulevard, Newark, NJ 07102, USA Universidad Nacional de Colombia Bogota Colombia; 2 Department of Ecology and Evolutionary Biology, University of Toronto, Toronto, ON M5S, Canada Department of Biological Sciences, New Jersey Institute of Technology Newark United States of America; 3 Instituto de Ciencias Naturales, Universidad Nacional de Colombia, Carrera 30 No. 45–03, Bogotá, D.C., Colombia University of Toronto Toronto Canada

**Keywords:** Colombian Amazon, *Metopiellus*, Staphylinidae

## Abstract

The genus *Metopiellus* (Staphylinidae, Pselaphinae) is confirmed in Colombia with the description of *Metopiellusguanano***sp. nov.** from the northern Amazon. Major diagnostic characters, a distributional map, and ecological data are given. Finally, a previous taxonomic key to *Metopiellus* is updated to include the new species.

## ﻿Introduction

Sakchoowong et al. (2008) stated that Pselaphinae beetles are ubiquitous, diverse, and poorly explored in the tropics. Thirty-nine Pselaphinae tribes show evidence of myrmecophily, and some are composed primarily or exclusively of myrmecophiles, such as Arnyliini, Attapseniini, Clavigerini, Colilodionini, Ctenistini, Metopiasini, Tiracerini, and Tmesiphorini (Parker 2016). The Neotropical genus *Metopiellus*Raffray 1908, of the tribe Metopiasini, currently consists of four species: *M.aglenus* Reitter, 1885, *M.hirtus* Reitter, 1885, and *M.painensis*Asenjo et al., 2017, described from Brazil, and *Metopiellussilvaticus* Bruch, 1933, known from Argentina. In this paper, we describe a new species of *Metopiellus* from the Northern Colombian Amazon (from the city of Mitu and the town of Villa Fatima in the Department of Vaupes). These records represent the first species-level documentation of *Metopiellus* in Colombia.

## ﻿Materials and methods

Samples were examined using a Leica Wild M3C stereo microscope. Z-stepped micrographs were captured using a Leica MC170 HD camera with a Leica 10450528 adapter (0.5x) camera mounted on a Leica M205 A microscope with a 1x objective. Dissections of the apical segments of the abdomen were made under a Motic SMZ-168 microscope (maximum magnification of 80x). The extracted segments were then cleared in a 10% KOH per weight solution for 20 minutes and rinsed in distilled water. Morphological character terminology, including foveation and nomenclature/initials, follows Chandler (2001) and Asenjo et al. (2019). Final plates were edited using Adobe Illustrator CS6 (Adobe Systems Inc., California, USA).

### ﻿Measurement abbreviations

**BL** body length (from margin of antennal tubercle of head to posterior margin of tergite VIII).

**BW** body width (maximum width of elytra).

**EL** elytral length (maximum).

**EW** elytral width (maximum).

**HL** head length (from anterior margin of antennal tubercle of head to posterior margin of head disc).

**HW** head width (maximum).

**NW** neck width (minimum).

**PL** pronotum length (maximum).

**PW** pronotum width (maximum, without spines).

### ﻿Repositories

Collections are referred to by the following acronyms:

**ICN**Instituto de Ciencias Naturales de la Universidad Nacional de Colombia, Bogotá, Colombia.

## ﻿Results

### ﻿ Metopiellus guanano sp. nov.

Taxon classificationAnimaliaColeopteraStaphylinidae

418FEC95-03E7-55D7-BA75-E8755969619B

http://zoobank.org/579DD444-A6E7-47A5-9394-5648733B4EAB

Figs 1 , 2 , 3

#### Type material

**(1 ♂, 1 ♀). *Holotype***: Colombia: 1 ♂: Vaupés department, Mitú, kilómetro 16 carretera vía Mitú-Monfort, Cucura. 1°08'41.6"N, 70°08'06.6"W. 10 Aug. 2019, Winkler 48 h. Col. Fernandez Lab. ICN 099808.

***Paratype***: Colombia: 1♀: Vaupés department, Villa Fatima, Pie de Cerro Tipiaca, 1°01'30.0"N, 69°58'37.2"W. 19 March. 2020, Winkler 40 h. Lote VW01. Col. Fernandez Lab. ICN 099807. Both the holotype and the paratype are deposited in ICN.

#### Diagnosis.

**Figure 1.**
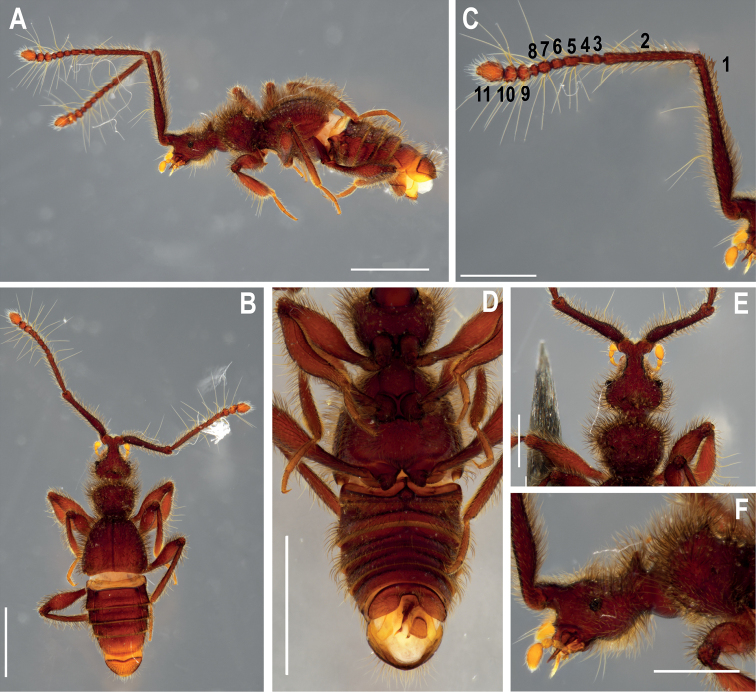
♂ *Metopiellusguanano* sp. nov., holotype **A** habitus, left lateral view **B** habitus, dorsal view **C** left antenna, lateral view **D** habitus, ventral view **E** head and pronotum, dorsal view **F** head and pronotum, left lateral view. Scale bars: 1 mm (**A, B, D**); 0.5 mm (**C, E, F**).

**Figure 2.**
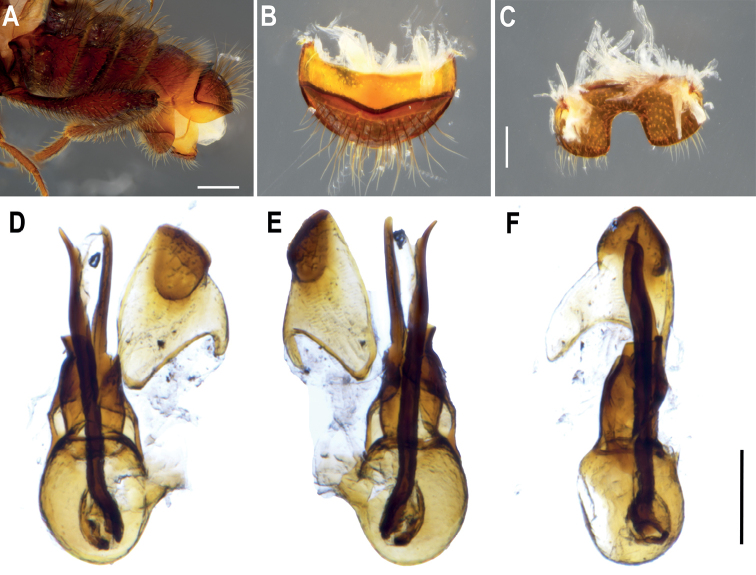
♂ *Metopiellusguanano* sp. nov., holotype **A** segment VIII (tergum VIII and sternum VIII), lateral view **B** sternum VIII **C** tergum VIII **D**, aedeagus, ventral view **E** aedeagus, dorsal view **F** aedeagus, lateral view. Scale bars: 0.5 mm (**A**); 0.2 mm (**B, C**); 0.2 mm (**D–F**).

*Metopiellusguanano* sp. nov. is most similar to *M.painensis*Asenjo et. al., 2017. Yet, it can be distinguished by the presence of a significant number of autapomorphic character states, such as: the presence of a prominent, horn-like spine on the vertexal region of the head (Figs 1A,F, 4A,B), the presence of 4 distinct pronotal spines and 2 deep elytral sulci, as well as the shape of the aedeagus (Fig. 2D–F) and thick pilosity covering the entire body (Fig. 1).

#### Description.

**Holotype male.** Body, mouthparts, antennae, and tarsi reddish light brown (Figs 1–2).

***Measurements***: BL (2.54 mm), BW (0.7 mm), EL (0.66 mm), EW (0.35 mm), HL (0.4 mm), HW (0.38 mm), NW (0.18 mm), PL (0.35 mm), PW (0.44 mm).

***Head*** (Figs 1E–F, 4A, B): pyriform (HL: 0.4 mm; HW: 0.38), anterior region distinctly narrower, raised at antennal tubercle. Antennal tubercule foveated and coarse. Posterior margin of head abruptly narrowed and with posterior-lateral angles rounded. Neck almost 2/3 width of head, lateral margins slightly obtuse (Fig. 1E). Head with two vertexal foveae [VF] (Fig. 1E) near posterior margin. Medial spine protruding from vertex between the vertexal foveae, similar to spines on pronotum. Vertex longitudinally impressed with sulcus running from anterior margin of antennal tubercle to vertexal fovea, branching out at level of eyes; sulcus narrow. Ventral surface of head with long, thin gular sulcus, interrupted at posterior third by two large gular foveae [GF]. Head covered in thick curved setae. Compound eyes small and slightly protruding laterally, composed of 12 ommatidia (Fig. 1F). Antennae (Fig. 1C) about 3/4 body length, scape almost half antennal length, last three antennomeres abruptly widened, scape length (all lengths without peduncle) 1.2 mm, width 0.15 mm, pedicel shorter than scape (length 0.59 mm: width 0.07), antennomeres 3–4 and 6–7 about as long as wide, antennomere 5 much longer than wide: 3 (length 0.07 mm: width 0.07 mm), 4 (length 0.06 mm: width 0.07 mm), 5 (length 0.11 mm: width 0.06 mm), 6 (length 0.08 mm: width 0.07 mm), 7 (length 0.08 mm: width 0.07 mm); antennomere 8 wider than long (length 0.04 mm: width 0.08 mm), antennomere 9 subcircular (length 0.08 mm: width 0.10 mm), antennomere 10 almost subquadrate (length 0.09 mm: width 0.11 mm), antennomere 11 longitudinally oval, with pointed apex (length 0.19 mm: width 0.13); all antennomeres with coarse integument and covered by long setae as well as thick, suberect pilosity.

***Thorax*** (Fig. 1B, D–F): pronotum trapezoidal in dorsal view (PL: 0.35; PW: 0.44) widest anteriorly, stair-shaped in profile. Two rounded protuberances on medial region of anterior half, acuminated with two spines. Two smaller spines produced laterally on each side of two rounded protuberances of medial region of anterior half of pronotum. Posterior half well below height of anterior half, demarcated by a deep sulcus connected to two deep, lateral antebasal foveae. Pronotum coarse, covered in thick, curved setae. Pronotum anterior margin slightly convex, basal margin straight. Prosternum with lateral procoxal fovea. Mesoventrite with prepectal fovea and lateral mesosternal fovea. Metaventrite with lateral mesocoxal foveae, a lateral metasternal fovea and a median metasternal fovea. Region of metaventrite in articulation with metacoxae forming a triangular protuberance, inwardly convex. “Waist” between pronotum and elytra strongly produced, with dark, coarsely reticulated integument.

***Elytra***: subquadrate (EL: 0.66; EW: 0.35), sides gradually broadening apically (Fig. 1A, B). Posterior margins convex, humeri without small longitudinal carina. Elytron uniformly rounded. No conspicuous basal elytral foveae (possibly replaced by sulci). Apicolateral margin of elytra slightly notched.

***Legs*** (Fig. 1A, D): long and robust. Femora thickened in apical half. Tibiae slightly curved and slightly shorter than femora, all tibiae thickened at apex. Protibiae carinate on inner surface and without microsetae on posterior and mesial regions, carinae lined with thick, curved setae. Tarsi 3-segmented, first tarsomeres very short, last 2 tarsomeres longer, tarsomere 2 longer than segment 3; all tarsi with single claw and thick accessory seta. Procoxae conical and prominent, mesocoxae globular-conical, less prominent than procoxae, metacoxae transverse, region that articulates with meta-trochanter conical. Procoxae, mesocoxae and metacoxae contiguous.

***Abdomen*** (Fig. 2A–C): slightly margined, with five visible tergites (morphological tergites IV–VIII), tergite VIII with rounded apex. Tergites and sternites IV–VII fused and bordered by a prominent carina. Sternite III visible as a small transverse plate between metacoxae, with long, transversal sulcus (Fig. 1D). Sternum IX divided longitudinally (Figs 1D, 2B).

***Aedeagus***: (Fig. 2D–F). Asymmetrical, with median lobe slightly bulbous at base, elongate and narrow, curved at apex. Apical lobe straight in dorsal view (Fig. 2E).

**Figure 3.**
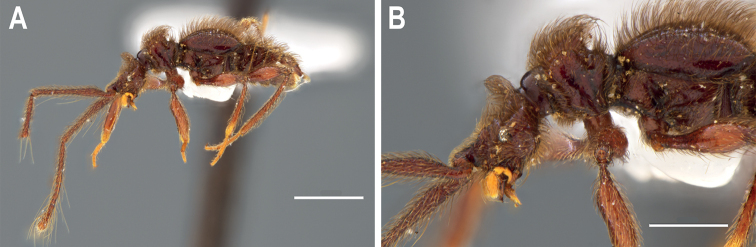
♀ *Metopiellusguanano* sp. nov., paratype **A** habitus, left lateral view **B** head and pronotum, left lateral view. Scale bars: 1 mm (**A**); 0.5 mm (**B**).

**Female** with characters of head, pronotum, and elytra as are described for male. Abdominal sternum VIII with posterior margin rounded and without a small prolongation (Fig. 3A, B).

#### Habitat and ecological notes.

The specimens were collected through Winkler sampling in primary forest in the northern Colombian Amazon. The sampled localities correspond to areas with a relative humidity of 84% and an average temperature of 28 °C; at both locations the vegetation was characteristic of a humid tropical forest. The processes that determine the diversity and floristic composition of the forests are not well known (Cano and Stevenson 2008). The sampling in Cucúra was carried out at no more than 20 m from a body of water; the area had been slightly disturbed by the elimination of plants from the understory and the terrain was humid due to recent rains.

Sampling at Villa Fátima was carried out in a submontane primary forest. The collection area was mostly pristine, with predominantly arboreal vegetation with little understory vegetation. This may be due to the superficial first granite layer of the hill (Tepui) (Gröger 2000). Specimens of the *Apterostigmapilosum* ant complex (Lattke 1997) were abundant in the same samples as the holotype and paratype of the new species. It is important to highlight this morphological similarity to *Apterostigma* species, but further studies are required to indicate any type of relationship between the new beetle and these ants.

#### Etymology.

The new species is named after the indigenous communities located at the type locality. The Guanano people inhabit the Vaupés River region of Colombia, from the Santa Cruz area below Mitú to Ibacaba in the lower Vaupés, near the border with Brazil (Stenzel 2007).

**Figure 4.**
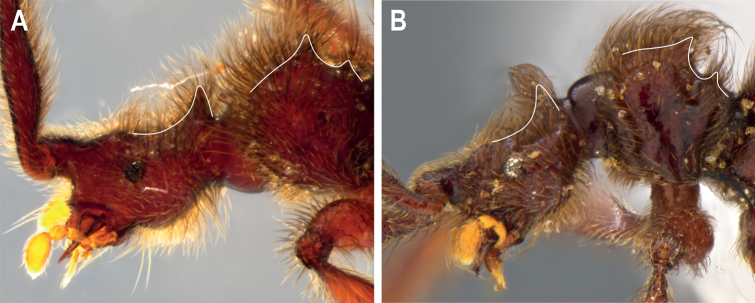
Detail of *Metopiellusguanano* sp. nov., head and pronotal spines, traced in white **A** male head and pronotum, left lateral view **B** female head and pronotum, left lateral view.

#### Distribution.

**Figure 5.**
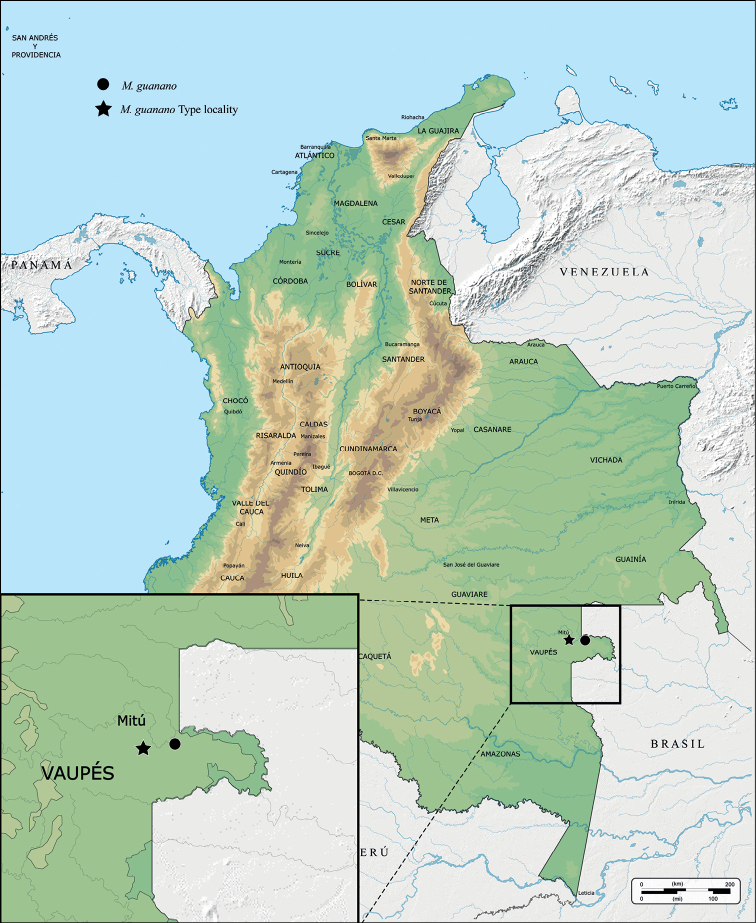
Geographic distribution of *Metopiellusguanano* sp. nov. The larger, filled black star denotes the type locality.

*Metopiellusguanano* sp. nov. is known from two localities: the counties of Mitu and Villa Fatima, Department of Vaupes, Colombia (Fig. 5).

#### Comments.

The new species belongs to the genus *Metopiellus* based on the shape of the third antennal segment, which is much shorter than the second (Fig. 1C), the posterior coxae contiguous or nearly so, and the mesial face of the protibia carinate and open at its base and apex (Fig. 1D) (Comellini 1983; Asenjo et al. 2017). However, the new species appears to be unique derived, presenting a horn-like spine on the vertexal margin of the head and spinose protrusions on the pronotum, as well as a medial protrusion on the dorsum of the pronotum (Figs 1F, 3B, 4A, B).

Newton et al. (2005) recorded the genus *Metopiellus* for the first time in Colombia, and Sissa and Navarrete (2016) also documented the genus in a study of the composition and structure of rove beetles in the department of Boyacá. However, neither of these studies identified species and we here provide the first species-level record of *Metopiellus*, and indeed of the tribe Metopiasini, from Colombia.

### ﻿Key to species of *Metopiellus* (based on Asenjo et al. 2017)

**Table:** 

1	Head with a horn-like spine protruding from the vertexal region; mesonotum with 2 acuminate bulbous projections	***Metopiellusguanano* sp. nov.**
–	Head simple, lacking a horn-like spine; mesonotum simple, without spines or projections	**2**
2	Head similar in width to pronotum; eyes absent	***Metopiellusaglenus* (Reitter)**
–	Head narrower than pronotum; eyes small or almost absent	**3**
3	Pedicel almost half the length of scape; antennomere 5 longer than combined length of antennomeres 3 and 4	***Metopielluspainensis* Asenjo et al.**
–	Pedicel less than half the length of scape; antennomere 5 shorter than combined length of antennomeres 3 and 4	**4**
4	Antennomere 8 transverse; eyes small	***Metopiellushirtus* (Reitter)**
–	Antennomere 8 obconical; eyes almost absent	***Metopiellussilvaticus* Bruch**

## Supplementary Material

XML Treatment for Metopiellus guanano
